# Supplementary figures and images for: Laboratory and molecular surveillance of paediatric typhoidal Salmonella in Nepal: Antimicrobial resistance and implications for vaccine policy
Source: PLoS Negl Trop Dis. 2018 Apr 23;12(4):e0006408. doi: 10.1371/journal.pntd.0006408 (PMC5933809; doi:10.1371/journal.pntd.0006408)

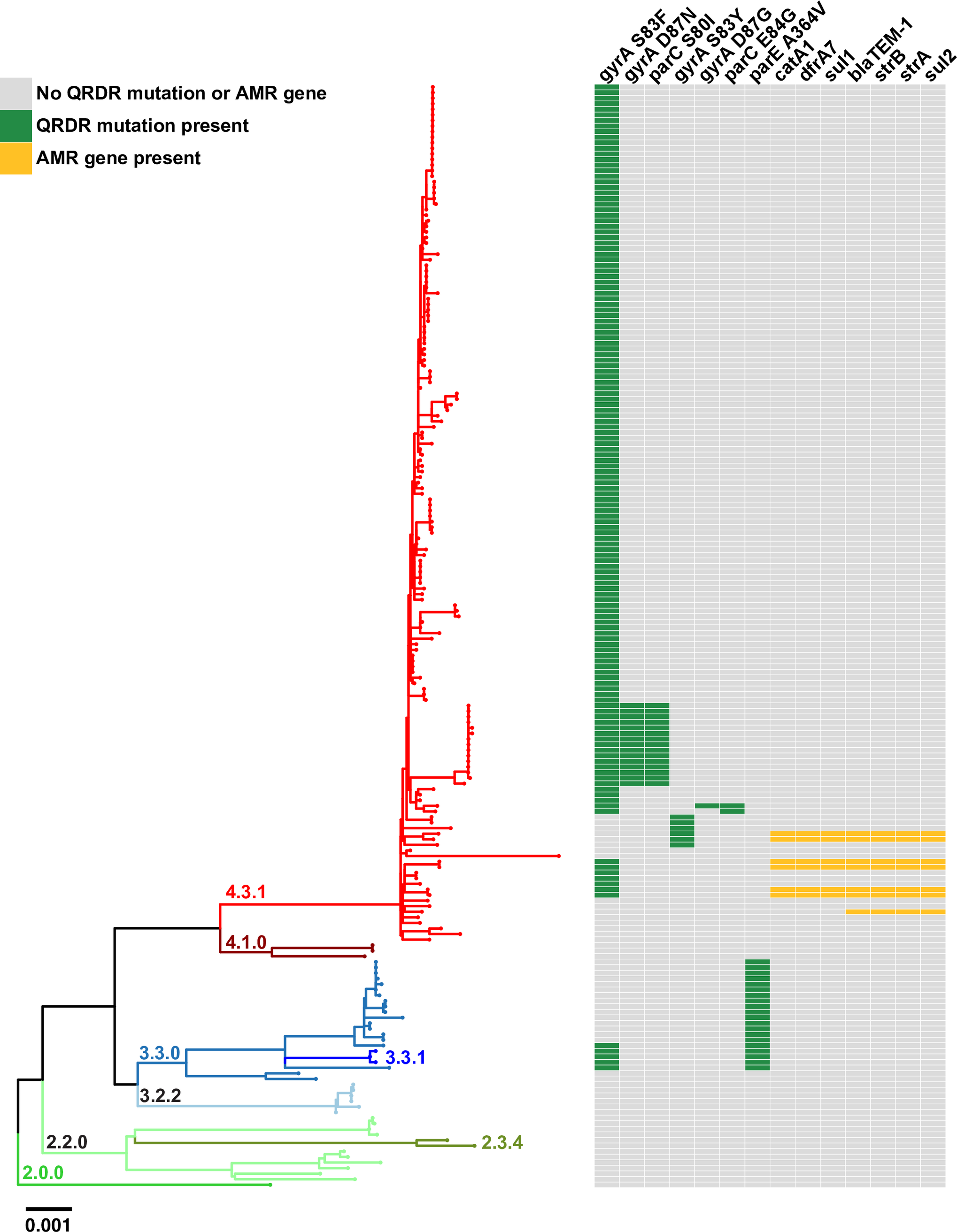

Supplement: S1 Fig — Paratyphi A outgroup rooted maximum likelihood phylogeny. Branch colours indicate the genotype (as labelled). Branch lengths are indicative of the estimated number of substitution rate per variable site. The adjacent heatmap shows the presence of AMR genes (yellow) and QRDR mutations (green) present in each isolate as described in the inset legend. (TIF) [file pntd.0006408.s001.tif]

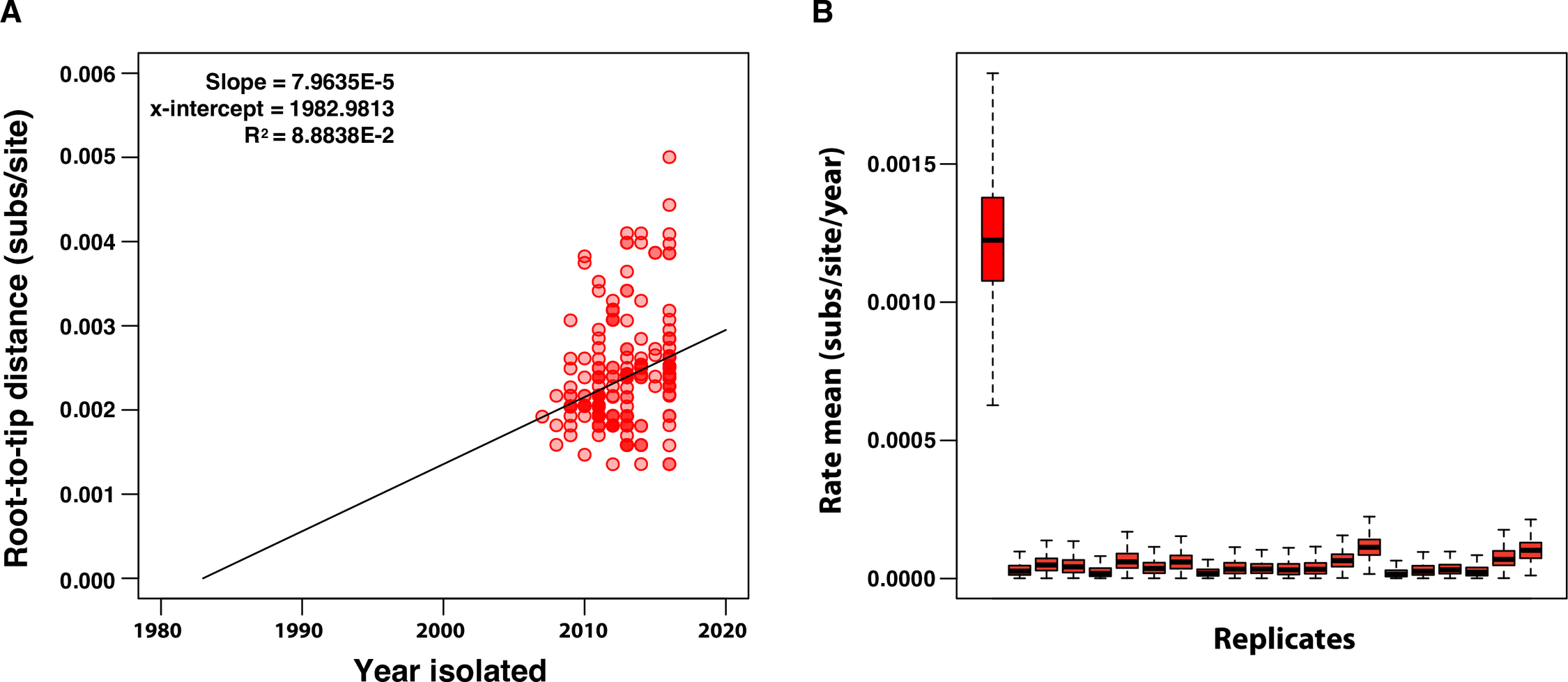

Supplement: S2 Fig — (A) Tempest regression of root-to-tip distance as (in the SNP alignment) a function of sampling time, with the root of the tree selected using heuristic residual mean squared (each point represents a tip of the maximum likelihood tree). The slope is a crude estimate of the substitution rate for the SNP alignment, the x-intercept corresponds to the age of the root node, and the R2 is a measure of clocklike behaviour (B) Date randomisation test with the left most box plot showing the posterior substitution rate estimate from the SNP alignment of the data with the correct sampling times, and the remaining 20 boxplots showing the posterior distributions of the rate from replicate runs using randomised dates. The data are considered to have strong temporal structure if the estimate with the correct sampling times does not overlap with those from the randomisations. (TIF) [file pntd.0006408.s002.tif]

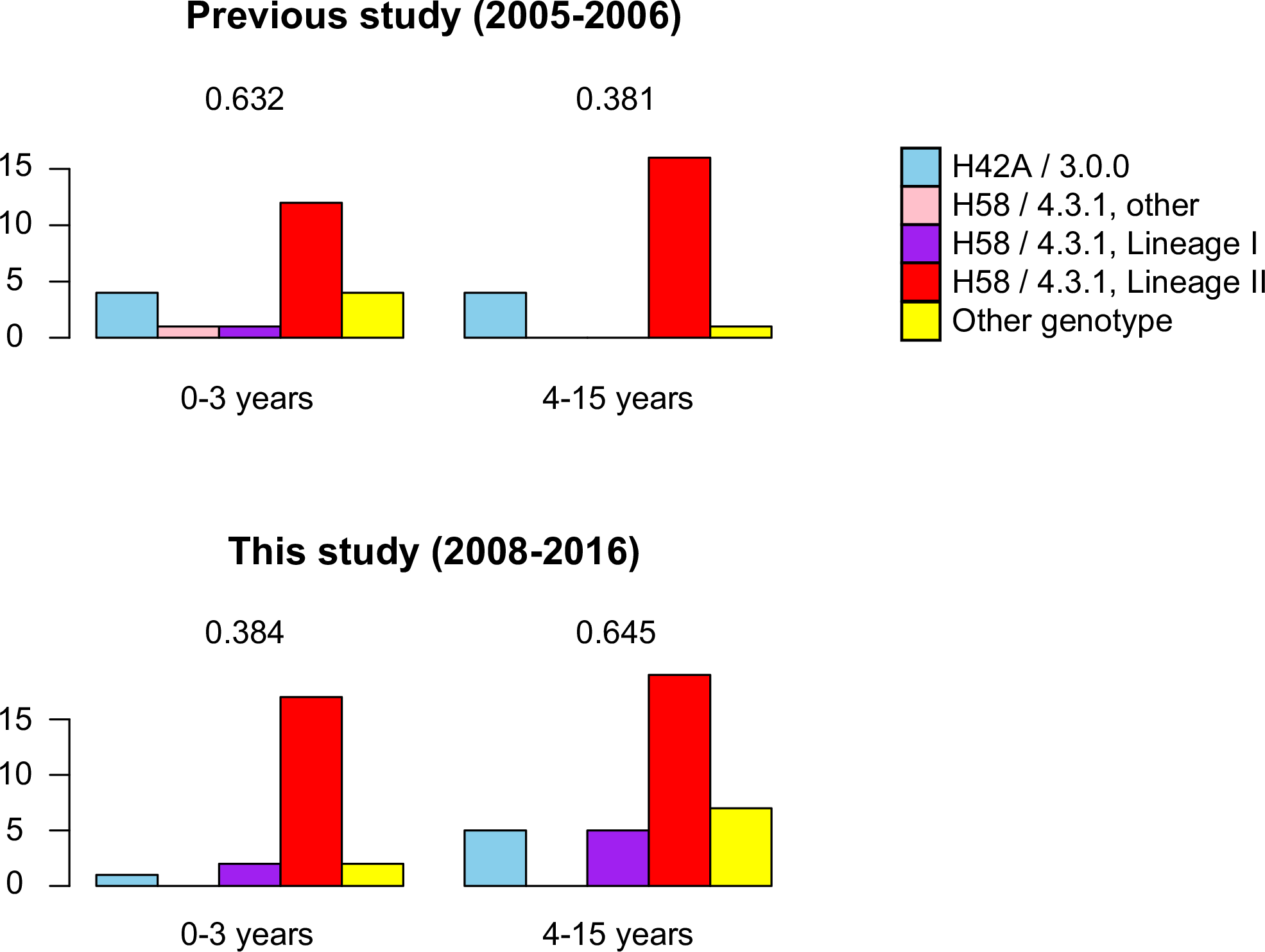

Supplement: S3 Fig — Simpson’s diversity of S. Typhi genotypes is printed on each plot. (TIF) [file pntd.0006408.s003.tif]
